# Dimethyl 2-(2,4,6-trimethoxy­benz­yl)malonate

**DOI:** 10.1107/S1600536810015928

**Published:** 2010-05-19

**Authors:** Shou-Xin Liu, Xin Lu, Shi-Rui Gao, Jian-Rong Han, Xiao-Li Zhen

**Affiliations:** a College of Chemical & Pharmaceutical Engineering, Hebei University of Science & Technology, Shijiazhuang 050018, People’s Republic of China; b College of Sciences, Hebei University of Science & Technology, Shijiazhuang 050018, People’s Republic of China

## Abstract

In the title compound, C_15_H_20_O_7_, the benzene ring makes dihedral angles of 69.17 (5) and 80.81 (4)° with the two side chains of malonate. The two malonate side chains comprising C/C/O/C atoms are oriented at right angles [86.26 (6)°] with respect to each other. In the crystal structure, the crystal packing is stabilized by weak non-classical inter­molecular C—H⋯O hydrogen bonds, which link the mol­ecules into an infinite network.

## Related literature

Substituted malonate, an important organic inter­mediate, is electrooxidized in methanol in the presence of halogen ions to afford the corresponding halomalonates, see: Okimoto & Takahashi (2002 ▶). For a related structure, see: Liu *et al.* (2010 ▶).
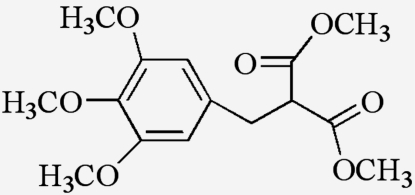


## Experimental

### 

#### Crystal data

C_15_H_20_O_7_
*M* *_r_* = 312.31
Monoclinic, 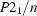

*a* = 11.6173 (15) Å
*b* = 8.1192 (10) Å
*c* = 17.236 (2) Å
β = 103.968 (2)°
*V* = 1577.7 (3) Å^3^
*Z* = 4
Mo *K*α radiation
μ = 0.11 mm^−1^
*T* = 296 K
0.28 × 0.24 × 0.20 mm

#### Data collection

Rigaku Saturn CCD area-detector diffractometer
Absorption correction: multi-scan (*CrystalClear*; Rigaku/MSC, 2005 ▶) *T*_min_ = 0.904, *T*_max_ = 0.935
9686 measured reflections
3851 independent reflections
2969 reflections with *I* > 2σ(*I*)
*R*_int_ = 0.019

#### Refinement

*R*[*F* ^2^ > 2σ(*F* ^2^)] = 0.040
*wR*(*F* ^2^) = 0.112
*S* = 1.05
3851 reflections
205 parameters
H-atom parameters constrained
Δρ_max_ = 0.21 e Å^−3^
Δρ_min_ = −0.15 e Å^−3^

### 

Data collection: *CrystalClear* (Rigaku/MSC, 2005 ▶); cell refinement: *CrystalClear*; data reduction: *CrystalClear*; program(s) used to solve structure: *SHELXS97* (Sheldrick, 2008 ▶); program(s) used to refine structure: *SHELXL97* (Sheldrick, 2008 ▶); molecular graphics: *SHELXTL* (Sheldrick, 2008 ▶); software used to prepare material for publication: *SHELXTL*.

## Supplementary Material

Crystal structure: contains datablocks I, global. DOI: 10.1107/S1600536810015928/pv2277sup1.cif

Structure factors: contains datablocks I. DOI: 10.1107/S1600536810015928/pv2277Isup2.hkl

Additional supplementary materials:  crystallographic information; 3D view; checkCIF report

## Figures and Tables

**Table 1:** Hydrogen-bond geometry (Å, °)

*D*—H⋯*A*	*D*—H	H⋯*A*	*D*⋯*A*	*D*—H⋯*A*
C5—H5b⋯O2^i^	0.96	2.54	3.353 (2)	142
C5—H5a⋯O5^ii^	0.96	2.57	3.503 (2)	164
C3—H3⋯O6^iii^	0.98	2.54	3.499 (2)	168

Symmetry codes: (i) 
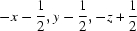
; (ii) 
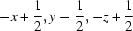
; (iii) 
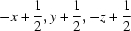
.

**Fig. 1.**
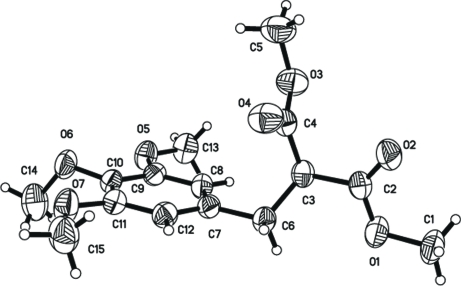
The title structure plotted with displacement ellipsoids at 50% probability level.

**Fig. 2.**
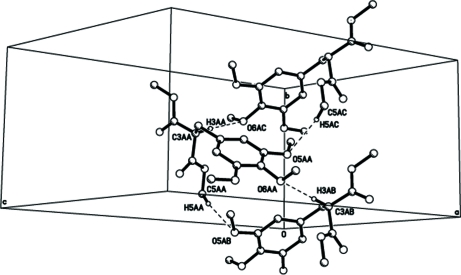
A unit cell showing intermolecular hydrogen bonds by dashed lines; the H-atoms not involved in H-bonds have been excluded for clarity.

## supplementary crystallographic information

### Comment

Substituted malnoate is a very important organic intermediate. It is electrooxidized in methanol in the presence of halogen ions to afford the corresponding halomalonates (Okimoto & Takahashi, 2002). We have synthesized dimethyl 2-(quinolin-methyene)malonate, and reported its structure (Liu *et al.*, 2010). We now report the synthesis and structure of the title compound, (I).

In the title compound ( Fig. 1), the benzene ring makes dihedral angles of 69.17 (5) and 80.81 (4)° with two side chains of malonate. In the crystal structure, the two malonate side chains comprising C/C/O/O atoms (C2/C3/O1/O2 and C113/C4/O3/C4) are oriented at right angles (86.26 (6)°) with respect to each other. In the crystal structure, the crystal packing is stabilized by weak non-classical intermolecular C—H···O hydrogen bonds which link the molecules into an infinite network; details have been provided in Table 1 and Fig. 2.

### Experimental

An anhydrous methanol solution (130 ml) of 1-brommethyl-3,4,5-trimethoxy (26.0 g, 0.1 mol) was added to an anhydrous methanol solution (180 ml) of sodium methoxide (5.4 g, 0.1 mol) and dimethyl malonate (26.4 g, 0.2 mol). The mixture was refluxed for 6 hours. The product was isolated with silica gel column, then the solvent was removed and added petroleum ether (5 ml) to the white precipitate. The precipitate was then isolated, recrystallized from n-hexane, and dried in a vacuum to give the title compound in 75% yield. Colorless single crystals of (I) suitable for X-ray analysis were obtained by slow evaporation of an n-hexane solution.

### Refinement

The H atoms were included in calculated positions (C—H = 0.93–0.98 Å) and refined as riding with *U_iso_*(H) = 1.2*U_eq_*(C) or 1.5*U_eq_*(methyl C).

### Crystal data

**Table:**

C_15_H_20_O_7_	*F*(000) = 664
*M**_r_* = 312.31	*D*_x_ = 1.315 Mg m^−^^3^
Monoclinic, *P*2_1_/*n*	Mo *K*α radiation, λ = 0.71073 Å
Hall symbol: -P 2yn	Cell parameters from 3043 reflections
*a* = 11.6173 (15) Å	θ = 2.4–24.5°
*b* = 8.1192 (10) Å	µ = 0.11 mm^−^^1^
*c* = 17.236 (2) Å	*T* = 296 K
β = 103.968 (2)°	Prism, colourless
*V* = 1577.7 (3) Å^3^	0.28 × 0.24 × 0.20 mm
*Z* = 4	

### Data collection

**Table:**

Rigaku Saturn CCD area-detector diffractometer	3851 independent reflections
Radiation source: fine-focus sealed tube	2969 reflections with *I* > 2σ(*I*)
graphite	*R*_int_ = 0.019
Detector resolution: 7.31 pixels mm^-1^	θ_max_ = 28.3°, θ_min_ = 1.9°
ω and φ scans	*h* = −14→12
Absorption correction: multi-scan (*CrystalClear*; Rigaku/MSC, 2005)	*k* = −8→10
*T*_min_ = 0.904, *T*_max_ = 0.935	*l* = −19→22
9686 measured reflections	

### Refinement

**Table:**

Refinement on *F*^2^	Secondary atom site location: difference Fourier map
Least-squares matrix: full	Hydrogen site location: inferred from neighbouring sites
*R*[*F*^2^ > 2σ(*F*^2^)] = 0.040	H-atom parameters constrained
*wR*(*F*^2^) = 0.112	*w* = 1/[σ^2^(*F*_o_^2^) + (0.0481*P*)^2^ + 0.3127*P*] where *P* = (*F*_o_^2^ + 2*F*_c_^2^)/3
*S* = 1.05	(Δ/σ)_max_ < 0.001
3851 reflections	Δρ_max_ = 0.21 e Å^−^^3^
205 parameters	Δρ_min_ = −0.15 e Å^−^^3^
0 restraints	Extinction correction: *SHELXL97* (Sheldrick, 2008), Fc^*^=kFc[1+0.001xFc^2^λ^3^/sin(2θ)]^-1/4^
Primary atom site location: structure-invariant direct methods	Extinction coefficient: 0.0172 (18)

### Special details

**Table:**

Geometry. All esds (except the esd in the dihedral angle between two l.s. planes) are estimated using the full covariance matrix. The cell esds are taken into account individually in the estimation of esds in distances, angles and torsion angles; correlations between esds in cell parameters are only used when they are defined by crystal symmetry. An approximate (isotropic) treatment of cell esds is used for estimating esds involving l.s. planes.
Refinement. Refinement of *F*^2^ against ALL reflections. The weighted *R*-factor wR and goodness of fit *S* are based on *F*^2^, conventional *R*-factors *R* are based on *F*, with *F* set to zero for negative *F*^2^. The threshold expression of *F*^2^ > σ(*F*^2^) is used only for calculating *R*-factors(gt) etc. and is not relevant to the choice of reflections for refinement. *R*-factors based on *F*^2^ are statistically about twice as large as those based on *F*, and *R*- factors based on ALL data will be even larger.

### Fractional atomic coordinates and isotropic or equivalent isotropic displacement parameters (Å^2^)

**Table:**

	*x*	*y*	*z*	*U*_iso_*/*U*_eq_	
C1	−0.13204 (15)	0.9367 (2)	0.43372 (11)	0.0635 (4)	
H1A	−0.2035	0.9372	0.3918	0.095*	
H1B	−0.1042	1.0476	0.4447	0.095*	
H1C	−0.1479	0.8893	0.4811	0.095*	
C2	−0.07201 (11)	0.68523 (16)	0.39019 (7)	0.0404 (3)	
C3	0.02668 (11)	0.59332 (15)	0.36509 (7)	0.0395 (3)	
H3	0.0329	0.6362	0.3131	0.047*	
C4	−0.01013 (11)	0.41471 (17)	0.35462 (8)	0.0421 (3)	
C5	−0.10242 (17)	0.2143 (2)	0.26180 (11)	0.0722 (5)	
H5A	−0.0371	0.1390	0.2762	0.108*	
H5B	−0.1390	0.2036	0.2058	0.108*	
H5C	−0.1595	0.1898	0.2922	0.108*	
C6	0.14750 (11)	0.61262 (17)	0.42412 (8)	0.0424 (3)	
H6A	0.1692	0.7282	0.4279	0.051*	
H6B	0.1413	0.5765	0.4766	0.051*	
C7	0.24446 (11)	0.51492 (15)	0.39988 (8)	0.0389 (3)	
C8	0.26120 (11)	0.52716 (16)	0.32288 (8)	0.0403 (3)	
H8	0.2142	0.5980	0.2861	0.048*	
C9	0.34815 (11)	0.43345 (15)	0.30108 (8)	0.0390 (3)	
C10	0.41922 (11)	0.32810 (16)	0.35617 (8)	0.0411 (3)	
C11	0.40304 (11)	0.31759 (16)	0.43313 (8)	0.0429 (3)	
C12	0.31541 (11)	0.41072 (16)	0.45482 (8)	0.0426 (3)	
H12	0.3044	0.4030	0.5064	0.051*	
C13	0.30064 (15)	0.5376 (2)	0.16826 (9)	0.0598 (4)	
H13A	0.2188	0.5060	0.1589	0.090*	
H13B	0.3260	0.5284	0.1194	0.090*	
H13C	0.3097	0.6494	0.1868	0.090*	
C14	0.61684 (16)	0.2734 (3)	0.35421 (15)	0.0899 (7)	
H14A	0.6255	0.3796	0.3318	0.135*	
H14B	0.6648	0.1946	0.3348	0.135*	
H14C	0.6419	0.2792	0.4114	0.135*	
C15	0.47068 (17)	0.2007 (2)	0.56275 (10)	0.0675 (5)	
H15A	0.4958	0.3047	0.5876	0.101*	
H15B	0.5227	0.1153	0.5893	0.101*	
H15C	0.3911	0.1778	0.5665	0.101*	
O1	−0.04280 (9)	0.84060 (12)	0.40905 (6)	0.0532 (3)	
O2	−0.16628 (8)	0.62656 (13)	0.39094 (6)	0.0558 (3)	
O3	−0.06001 (10)	0.38021 (14)	0.27852 (6)	0.0579 (3)	
O4	0.00316 (11)	0.31615 (13)	0.40747 (6)	0.0613 (3)	
O5	0.37059 (9)	0.43260 (12)	0.22680 (6)	0.0505 (3)	
O6	0.49714 (9)	0.22459 (13)	0.33151 (6)	0.0539 (3)	
O7	0.47421 (10)	0.20690 (14)	0.48168 (6)	0.0623 (3)	

### Atomic displacement parameters (Å^2^)

**Table:**

	*U*^11^	*U*^22^	*U*^33^	*U*^12^	*U*^13^	*U*^23^
C1	0.0574 (9)	0.0518 (9)	0.0869 (12)	0.0155 (7)	0.0283 (8)	−0.0038 (8)
C2	0.0369 (6)	0.0443 (7)	0.0404 (6)	0.0060 (5)	0.0103 (5)	0.0068 (5)
C3	0.0389 (6)	0.0423 (7)	0.0400 (6)	0.0047 (5)	0.0150 (5)	0.0049 (5)
C4	0.0364 (6)	0.0462 (7)	0.0450 (7)	0.0040 (5)	0.0122 (5)	0.0005 (6)
C5	0.0692 (11)	0.0745 (12)	0.0693 (11)	−0.0141 (9)	0.0099 (9)	−0.0216 (9)
C6	0.0380 (6)	0.0429 (7)	0.0484 (7)	0.0028 (5)	0.0147 (5)	−0.0035 (6)
C7	0.0342 (6)	0.0365 (6)	0.0473 (7)	−0.0008 (5)	0.0125 (5)	−0.0037 (5)
C8	0.0371 (6)	0.0375 (6)	0.0481 (7)	0.0036 (5)	0.0136 (5)	0.0034 (5)
C9	0.0380 (6)	0.0365 (6)	0.0451 (7)	−0.0021 (5)	0.0150 (5)	−0.0022 (5)
C10	0.0377 (6)	0.0360 (6)	0.0505 (7)	0.0036 (5)	0.0124 (5)	−0.0061 (5)
C11	0.0420 (7)	0.0383 (7)	0.0467 (7)	0.0038 (5)	0.0078 (5)	−0.0010 (5)
C12	0.0432 (7)	0.0438 (7)	0.0422 (7)	0.0014 (5)	0.0129 (5)	−0.0018 (5)
C13	0.0678 (10)	0.0642 (10)	0.0507 (8)	0.0134 (8)	0.0206 (7)	0.0094 (7)
C14	0.0486 (10)	0.1037 (16)	0.1212 (18)	0.0205 (10)	0.0282 (10)	−0.0129 (13)
C15	0.0708 (11)	0.0756 (12)	0.0525 (9)	0.0189 (9)	0.0081 (8)	0.0119 (8)
O1	0.0482 (5)	0.0417 (5)	0.0760 (7)	0.0066 (4)	0.0271 (5)	0.0019 (5)
O2	0.0393 (5)	0.0585 (6)	0.0730 (7)	0.0012 (4)	0.0202 (5)	−0.0026 (5)
O3	0.0628 (6)	0.0628 (7)	0.0462 (6)	−0.0067 (5)	0.0098 (5)	−0.0055 (5)
O4	0.0764 (8)	0.0473 (6)	0.0553 (6)	−0.0060 (5)	0.0061 (5)	0.0080 (5)
O5	0.0555 (6)	0.0529 (6)	0.0487 (5)	0.0127 (4)	0.0238 (4)	0.0057 (4)
O6	0.0521 (6)	0.0520 (6)	0.0598 (6)	0.0174 (4)	0.0176 (5)	−0.0061 (5)
O7	0.0716 (7)	0.0625 (7)	0.0521 (6)	0.0302 (6)	0.0134 (5)	0.0090 (5)

### Geometric parameters (Å, °)

**Table:**

C1—O1	1.4411 (17)	C8—C9	1.3869 (17)
C1—H1A	0.9600	C8—H8	0.9300
C1—H1B	0.9600	C9—O5	1.3668 (15)
C1—H1C	0.9600	C9—C10	1.3915 (18)
C2—O2	1.1973 (16)	C10—O6	1.3756 (15)
C2—O1	1.3262 (17)	C10—C11	1.3864 (19)
C2—C3	1.5159 (17)	C11—O7	1.3630 (16)
C3—C4	1.5104 (19)	C11—C12	1.3898 (18)
C3—C6	1.5298 (18)	C12—H12	0.9300
C3—H3	0.9800	C13—O5	1.4175 (18)
C4—O4	1.1941 (16)	C13—H13A	0.9600
C4—O3	1.3296 (16)	C13—H13B	0.9600
C5—O3	1.439 (2)	C13—H13C	0.9600
C5—H5A	0.9600	C14—O6	1.408 (2)
C5—H5B	0.9600	C14—H14A	0.9600
C5—H5C	0.9600	C14—H14B	0.9600
C6—C7	1.5165 (17)	C14—H14C	0.9600
C6—H6A	0.9700	C15—O7	1.4086 (19)
C6—H6B	0.9700	C15—H15A	0.9600
C7—C12	1.3839 (18)	C15—H15B	0.9600
C7—C8	1.3907 (18)	C15—H15C	0.9600
			
O1—C1—H1A	109.5	O5—C9—C8	124.92 (12)
O1—C1—H1B	109.5	O5—C9—C10	114.85 (11)
H1A—C1—H1B	109.5	C8—C9—C10	120.22 (12)
O1—C1—H1C	109.5	O6—C10—C11	120.62 (12)
H1A—C1—H1C	109.5	O6—C10—C9	119.40 (12)
H1B—C1—H1C	109.5	C11—C10—C9	119.70 (11)
O2—C2—O1	123.81 (12)	O7—C11—C10	115.20 (11)
O2—C2—C3	124.33 (12)	O7—C11—C12	124.66 (12)
O1—C2—C3	111.83 (11)	C10—C11—C12	120.08 (12)
C4—C3—C2	107.15 (10)	C7—C12—C11	120.16 (12)
C4—C3—C6	111.45 (10)	C7—C12—H12	119.9
C2—C3—C6	113.29 (10)	C11—C12—H12	119.9
C4—C3—H3	108.3	O5—C13—H13A	109.5
C2—C3—H3	108.3	O5—C13—H13B	109.5
C6—C3—H3	108.3	H13A—C13—H13B	109.5
O4—C4—O3	123.82 (13)	O5—C13—H13C	109.5
O4—C4—C3	124.86 (12)	H13A—C13—H13C	109.5
O3—C4—C3	111.32 (11)	H13B—C13—H13C	109.5
O3—C5—H5A	109.5	O6—C14—H14A	109.5
O3—C5—H5B	109.5	O6—C14—H14B	109.5
H5A—C5—H5B	109.5	H14A—C14—H14B	109.5
O3—C5—H5C	109.5	O6—C14—H14C	109.5
H5A—C5—H5C	109.5	H14A—C14—H14C	109.5
H5B—C5—H5C	109.5	H14B—C14—H14C	109.5
C7—C6—C3	112.73 (10)	O7—C15—H15A	109.5
C7—C6—H6A	109.0	O7—C15—H15B	109.5
C3—C6—H6A	109.0	H15A—C15—H15B	109.5
C7—C6—H6B	109.0	O7—C15—H15C	109.5
C3—C6—H6B	109.0	H15A—C15—H15C	109.5
H6A—C6—H6B	107.8	H15B—C15—H15C	109.5
C12—C7—C8	119.96 (11)	C2—O1—C1	115.31 (11)
C12—C7—C6	119.38 (11)	C4—O3—C5	116.19 (13)
C8—C7—C6	120.66 (11)	C9—O5—C13	117.23 (11)
C9—C8—C7	119.87 (12)	C10—O6—C14	114.95 (12)
C9—C8—H8	120.1	C11—O7—C15	118.30 (12)
C7—C8—H8	120.1		

### Hydrogen-bond geometry (Å, °)

**Table:**

*D*—H···*A*	*D*—H	H···*A*	*D*···*A*	*D*—H···*A*
C5—H5b···O2^i^	0.96	2.54	3.353 (2)	142
C5—H5a···O5^ii^	0.96	2.57	3.503 (2)	164
C3—H3···O6^iii^	0.98	2.54	3.499 (2)	168

Symmetry codes: (i) −*x*−1/2, *y*−1/2, −*z*+1/2; (ii) −*x*+1/2, *y*−1/2, −*z*+1/2; (iii) −*x*+1/2, *y*+1/2, −*z*+1/2.
